# Clinicopathological and prognostic significance of long noncoding RNA MALAT1 in human cancers: a review and meta-analysis

**DOI:** 10.1186/s12935-018-0606-z

**Published:** 2018-08-06

**Authors:** Juan Li, Zhigang Cui, Hang Li, Xiaoting Lv, Min Gao, Zitai Yang, Yanhong Bi, Ziwei Zhang, Shengli Wang, Baosen Zhou, Zhihua Yin

**Affiliations:** 1 Department of Epidemiology, School of Public Health, China Medical University, No. 77 Puhe Road, Shenyang North New Area, Shenyang, 110122 People’s Republic of China; 2 Key Laboratory of Cancer Etiology and Intervention, University of Liaoning Province, Shenyang, 110122 People’s Republic of China; 3 School of Nursing, China Medical University, Shenyang, 110122 China

**Keywords:** LncRNA, MALAT1, Carcinoma, Prognosis, Meta-analysis

## Abstract

**Background:**

The aberrant regulation of MALAT1 has been indicated to be involved in various carcinogenic pathways contributing to the tumourigenesis and progression of cancers. The current meta-analysis summarized the research advances of MALAT1 functions and analyzed its prognostic value among multiple types of cancers.

**Methods:**

Eligible studies were identified through retrieving the PubMed, Web of Science, and CNKI databases, up to Mar 1, 2018. 28 studies of 5436 patients and 36 studies of 3325 patients were enrolled in the meta-analysis to evaluate the association of MALAT1 expression with survival outcomes and clinical parameters.

**Results:**

The results demonstrated that over-expression of MALAT1 may predict lymph node metastasis (pooled OR = 2.335, 95% CI 1.606–3.395, P = 0.000) and distant metastasis (pooled OR = 2.456, 95% CI 1.407–4.286, P = 0.002). Moreover, MALAT1 was also related with tumour size (pooled OR = 1.875, 95% CI 1.257–2.795, P = 0.002) and TNM stage (pooled OR = 2.034, 95% CI 1.111–3.724, P = 0.021). Additionally, elevated MALAT1 expression could predict poor OS (pooled HR = 2.298, 95% CI 1.953–2.704, P = 0.000), DFS (pooled HR = 2.036, 95% CI 1.240–3.342, P = 0.005), RFS (pooled HR = 2.491, 95% CI 1.505–4.123, P = 0.000), DSS (pooled HR = 2.098, 95% CI 1.372–3.211, P = 0.001) and PFS (pooled HR = 1.842, 95% CI 1.138–2.983, P = 0.013) in multivariate model. Importantly, subgroup analyses disclosed that increased MALAT1 expression had a poor OS among different cancer types (Estrogen-dependent cancer: pooled HR = 2.656, 95% CI 1.560–4.523; urological cancer: pooled HR = 1.952, 95% CI 1.189–3.204; glioma: pooled HR = 2.315, 95% CI 1.643–3.263; digestive cancer: pooled HR = 2.451, 95% CI 1.862–3.227).

**Conclusions:**

The present findings demonstrated that MALAT1 may be a novel biomarker for predicting survival outcome, lymph node metastasis and distant metastasis.

**Electronic supplementary material:**

The online version of this article (10.1186/s12935-018-0606-z) contains supplementary material, which is available to authorized users.

## Background

Long non-coding RNAs (lncRNAs) as genomic “dark matters” have been disclosed to be closely related to the development of cancer [1–3], which lead to the alteration of oncogenic phenotypes including cell proliferation, differentiation, metastasis, apoptosis and invasion [3–6]. Based on the current emerging evidence, cancer-related lncRNAs may be candidate biomarkers for affording precise diagnosis, appraisal of personalized prognosis, evaluation of targeted therapy and prediction of lymph node metastasis, distant metastasis as well as tumour differentiation [7–9].

The metastasis-associated lung adenocarcinoma transcript 1 (MAlAT1) is mapped to human chromosome 11q13 [10, 11]. Differentiating from other members of lncRNAs family, MALAT1 is a broadly expressed and evolutionarily conserved lncRNA with length of more than 8000 nt. Ji et al. initially discovered that MALAT1, a metastasis-associated gene, may be used to identify early-stage NSCLC patients that are at high risk to develop deterioration with metastasis [12]. Evidence for the carcinogenic roles of MALAT1 has gradually emerged from previous fundamental and clinical studies. For example, MALAT1 silencing might impede proliferation, migration, and invasion of triple-negative breast cancer (TNBC) cell by reversely mediating MiR-129-5p [13]. MALAT1 acts as a competitive endogenous RNA (ceRNA) to regulate ZEB1 expression by sponging miR-143-3p, whereas miR-143-3p inhibitor partially impaired the effect of MALAT1 on hepatocellular carcinoma (HCC) cells, and the inhibition of MALAT1 also might inhabit proliferation and invasion of HCC cells [14]. Moreover, activation of p53 may be due to depletion of MALAT1, which may result in cell cycle defects that are sensitive to p53 levels [15]. MALAT1 expression might be regulated by DNA methylation in lung cancer by evaluating methylation status of the CpG island at the MALAT1 promoter [16]. Furthermore, up-regulated MALAT1 promotes cell metastasis by activating the Wnt/β-catenin signaling pathway to promote EMT of bladder cancer cells [17, 18]. Transition of oncogenic phenotypes caused by MALAT1 have also been observed in cervical cancer [19], gastric cancer [20], prostate cancer [21]. Taken together, emerging evidence manifested that dysregulated MATAT1 is closely related to the development of various types of cancer.

For the recent decade, increasing studies have demonstrated the influence of MALAT1 expression on clinicopathological parameters and prognostic outcomes among diffident types of cancer, including digestive cancers [20, 22–26], gliomas [27, 28], estrogen-dependent cancers [29–31], urological cancers [32–34] and other cancers [12, 35]. However, these published studies have emerged the inconsistent and controversial conclusions [22, 32, 34, 36]. Herein, we conducted a systematic review and meta-analysis to elucidate the relationship of MALAT1 with prognosis or clinical features and generalized its tumorigenicity among different cancers.

## Materials and methods

### Literature search

Eligible records were systematically retrieved in three authoritative databases including PubMed, Web of Science, and CNKI databases up to March 1, 2018 to obtain relevant articles regarding prognostic and clinicopathological outcomes of MALAT1 among malignant cancers, with the following keywords including “MALAT1 expression and (outcome or prognosis or prognostic or mortality or survival) and (cancer OR carcinoma OR tumor OR malignancy OR neoplasm OR lymphoma OR leukemia)”. Besides, the references lists of included studies were retrieved to guarantee that all qualified studies contained in the pooling analysis.

### Study selection and data extraction

Data extraction of each qualified articles was as follows: first author, year, country, ethnicity, type of cancers, follow-up (months), detection method, sample size, survival outcome and the corresponding HR and 95% CI and other data for clinical parameters. Eligible articles need to meet the following criteria: (a) studies with cancers diagnosed by pathological and histological confirmation; (b) studies with the survival outcomes such as “overall survival, “disease-free survival”, “recurrence-free survival”, “disease-specific survival”, “progression-free survival”, recurrence and mortality, and other clinical parameters such as lymph node metastasis, distant metastasis, differentiation/histological grade, tumor size and TNM stage; (c) original studies detected MALAT1 expression in tissue or plasma; (d) studies did explicitly provide HR and 95% CI. However, ineligible articles were excluded on the basis of the following criteria: (a) studies focused on other lncRNAs, diagnosis, polymorphism, case reports, reviews and meta-analyses; (b) studies did not provide available data; (c) studies only with mechanisms of MALAT1 and other genes; (d) animal studies of MALAT1 and other lncRNAs; (e) duplicated published reports, articles or data.

### Quality assessment

Two investigators individually assessed the quality of all included studies according to the Newcastle–Ottawa Scale (NOS), and the scale totally comprises subject selection, comparability of study groups as well as ascertainment of survival outcomes. Articles with NOS ≥ 6 scores were regard as high-quality studies.

### Statistical analysis

Cochran’s Q and I^2^ tests were applied to find the heterogeneity across studies. Hazard ratios (HRs), odds ratios (ORs) and their 95% confidence intervals (95% CIs) were calculated by using a random effect model when I^2^ > 50% and the corresponding P value < 0.05. Otherwise, a fixed effect model was used to estimate the pooled results. Subgroup analysis were further performed to find the source of heterogeneity. Each single study on the overall effect of the stability of the pooled results was estimated by performing sensitivity analyses. Egger’s test and Begg’s funnel plot were applied to identify publication bias. All calculated results of the meta-analysis were performed by using Stata 11 software. A P value < 0.05 was consistently regarded as statistical significance.

## Results

### Identification of the included studies

**Fig. 1:**
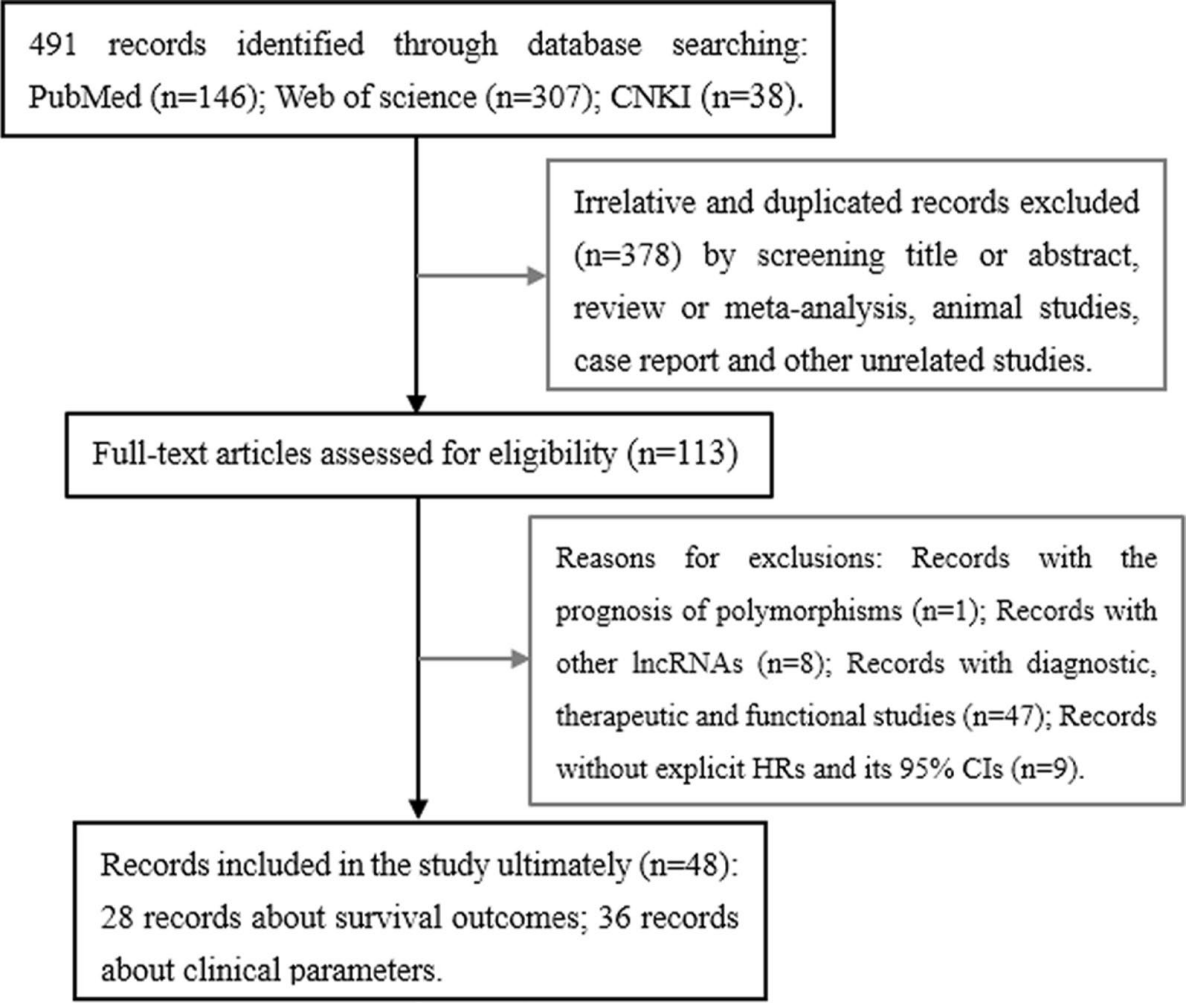
Flow diagram of articles and studies selection process

In the study, the detailed selection process of all 48 included articles presented in Fig. 1. A total of 5436 patients from 28 articles covering 54 cohort studies were included to evaluate prognostic value (presented in Additional file 1: Table S1, Additional file 2: Table S2). Of 54 studies with survival outcomes including OS, DFS, RFS, PFS and DSS, 25 studies from 19 articles [20, 22–24, 27, 29, 32, 34, 37–48] in univariate analysis, 29 studies from 21 articles [14, 23, 27–29, 32, 34, 35, 37, 39, 41–47, 49–52] in multivariate analysis. Additionally, 3325 patients from 36 articles [13, 17, 20, 23–27, 31–34, 37, 42–44, 46–50, 53–67] with clinical parameters including age, gender, lymph node metastasis (LNM), distant metastasis, differentiation, tumor size and TNM stage were enrolled in the study (data shown in Additional file 3: Table S3). The study contains four cancer types including digestive cancers with gastric cancer (GC), gallbladder cancer (GBC), esophageal cancer (EC), pancreatic duct adenocarcinoma (PDAC), esophageal squamous cell carcinoma (ESCC), hepatocellular carcinoma (HCC) and colorectal cancer (CRC); gliomas with glioblastoma, glioma and glioblastoma multiforme (GBM); estrogen-dependent cancers with cervical cancer (CC), epithelial ovarian cancer (EOC) and breast cancer (BC); and urological cancers with urothelial carcinoma (UC), bladder cancer and clear cell renal cell carcinoma (ccRCC). MALAT expression was detected by quantitative real time PCR (qRT-PCR) and in situ hybridization (ISH).

### Association of MALAT1 with clinicopathological parameters

**Fig. 2:**
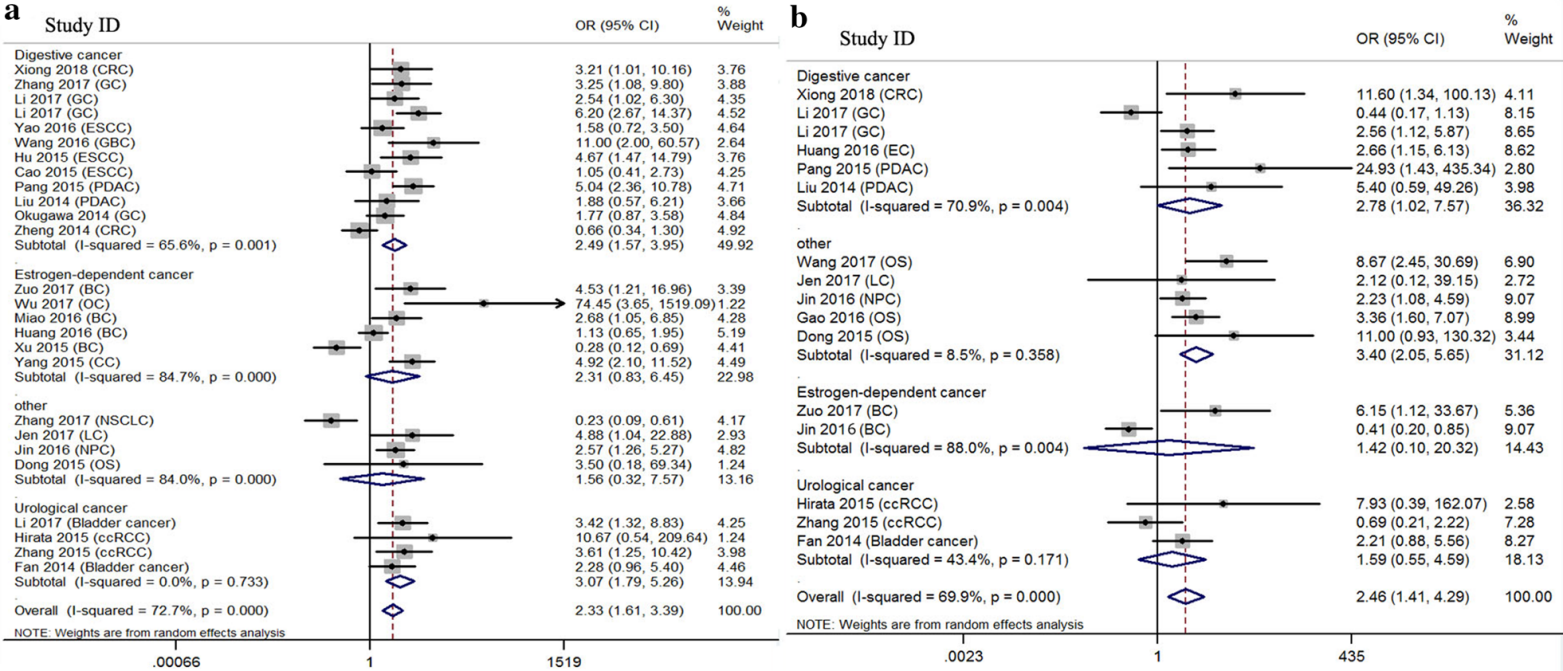
Forest plots of clinicopathological parameters stratified by cancer type. **a** Lymph node metastasis; **b** distant metastasis

As presented in Table 1, the significant association of MALAT1 expression with patients’ age or gender did not existed (age: P = 0.823 and gender: P = 0.080). The increased expression level of MALAT1 was significantly associated with lymph node metastasis (pooled OR = 2.335, 95% CI 1.606–3.395, P = 0.000), tumour size (pooled OR = 1.875, 95% CI 1.257–2.795, P = 0.002), distant metastasis (pooled OR = 2.456, 95% CI 1.407–4.286, P = 0.002) and TNM stage (pooled OR = 2.034, 95% CI 1.111–3.724, P = 0.021). Moreover, subgroup analysis of cancer type presented that patients with MALAT1 over-expression had higher risk of lymph node metastasis and distant metastasis (shown in Fig. 2a, b).

**Table 1:** Association between MALAT1 and clinicopathological parameters

Clinicopathological parameters	Studies (n)	Patient s (n)	OR (95% CI)	P	Heterogeneity (I^2^, P)	Model
Age (elderly vs. nonelderly)	33	3127	0.983 (0.848–1.140)	0.823	0.0%, 0.991	Fixed
Gender (female vs. male)	28	2459	0.860 (0.726–1.018)	0.080	12.6%, 0.275	Fixed
Tumor size (large size vs. small size)	19	1811	1.875 (1.257–2.795)	0.002	72.8%, 0.000	Random
Lymph node metastasis (positive vs. negative)	26	2440	2.335 (1.606–3.395)	0.000	72.7%, 0.000	Random
Distant metastasis (presence vs. absence)	16	1514	2.456 (1.407–4.286)	0.002	69.9%, 0.000	Random
Differentiation (poor vs. well, moderate)	21	1980	1.112 (0.916–1.351)	0.284	49.6%, 0.005	Fixed
TNM stage (III + IV vs. I + II)	11	1083	2.034 (1.111–3.724)	0.021	77.7%, 0.000	Random

If I^2^ > 50%, the results were calculated by random model

*OR* odds ratio, *CI* confidence interval

### Association of MALAT1 with survival outcomes

**Fig. 3:**
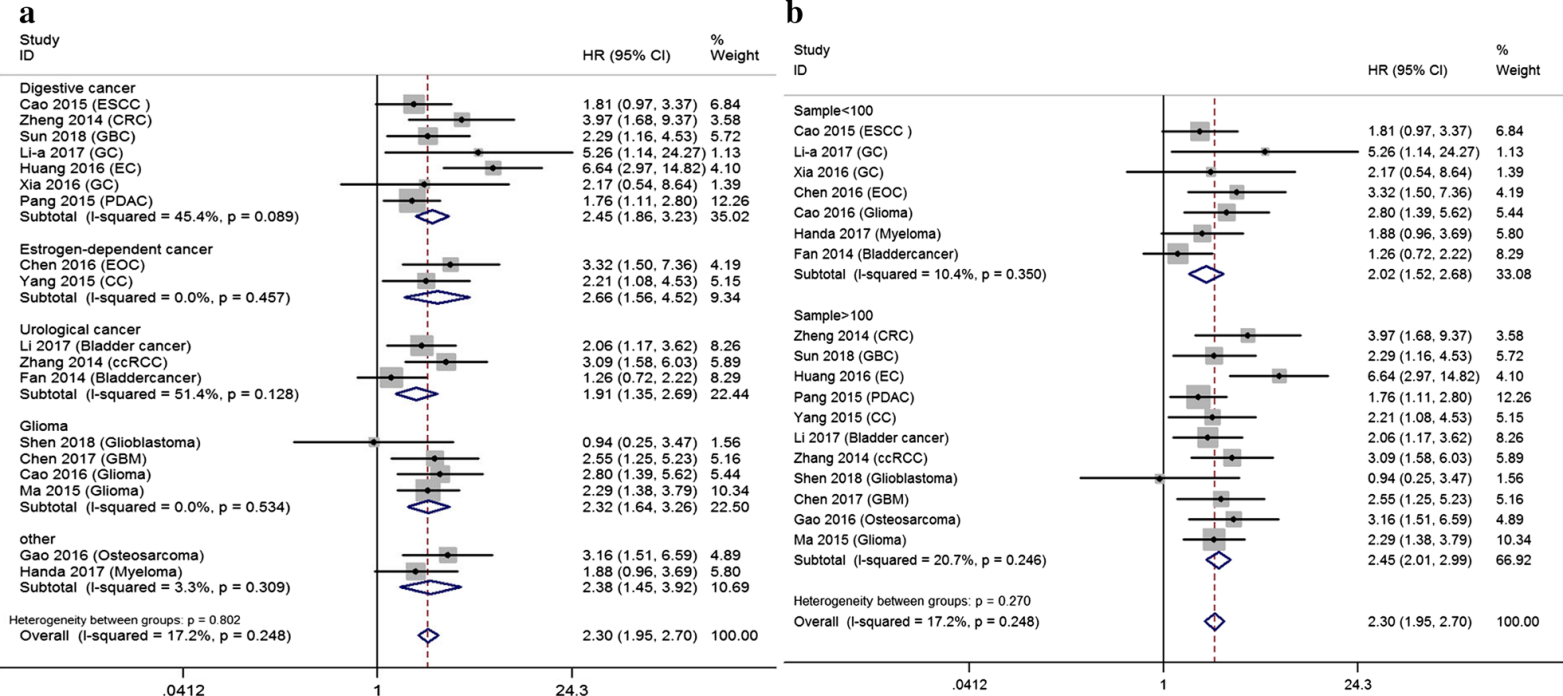
Forest plots of subgroup analysis of pooled HRs of OS in multivariate model. **a** Cancer type; **b** sample size

A total of 15 eligible studies with 1869 cases focused on evaluating the association between MALAT1 expression and overall survival in univariate model (Table 2). Overall, patients with elevated expression of MALAT1 had a poor OS in univariate (pooled HR = 2.296, 95% CI 1.716–3.072, P = 0.000) analysis with heterogeneity (I^2^ = 67.6%). Unlike subgroup of univariate analysis, almost all analytical results of multivariate subgroup had no significant heterogeneity (I^2^ < 50%). 18 studies comprising 1891 patients reported the relationship of MALAT1 expression with OS in multivariate analysis. Overexpression of MALAT1 had a higher risk of poor OS (pooled HR = 2.298, 95% CI 1.953–2.704, P = 0.000, I^2^ = 17.2%). MALAT1 high expression was also indicated to predict poor OS among different cancer types (Estrogen-dependent cancer: pooled HR = 2.656, 95% CI = 1.560–4.523; Urological cancer: pooled HR = 1.952, 95% CI 1.189–3.204; Glioma: pooled HR = 2.315, 95% CI 1.643–3.263; Digestive cancer: pooled HR = 2.451, 95% CI 1.862–3.227) (data shown in Table 2 and Fig. 3a). Subgroup analysis of sample size presented in Fig. 3b.

**Table 2:** Association between MALAT1 expression and overall survival

Survival analysis	Subgroup	Studies (n)	Patients (n)	HR (95% CI)	P	Heterogeneity (I^2^, P)
Univariate	Overall	15	1869	2.296 (1.716–3.072)	0.000	67.6%, 0.000
	Region					
	China	12	1209	2.793 (2.310–3.376)	0.000	0.0%, 0.924
	Other	3	660	1.164 (0.577–2.346)	0.672	83.5%, 0.002
	Cancer type					
	Estrogen-dependent carcinoma	2	198	3.296 (2.027–5.358)	0.000	22.9%, 0.255
	Urological carcinoma	2	226	3.184 (2.072–4.892)	0.000	0.0%, 0.739
	Glioma	2	258	2.750 (1.839–4.111)	0.000	0.0%, 0.553
	Digestive carcinoma	6	633	1.750 (0.974–3.143)	0.061	79.7%, 0.000
	Other	3	554	2.154 (1.471–3.154)	0.000	0.0%, 0.484
	Sample size					
	Sample < 100	5	333	2.443 (1.655–3.608)	0.000	0.0%, 0.752
	Sample > 100	10	1536	2.203 (1.516–3.202	0.000	77.9%, 0.000
Multivariate	Overall	18	1891	2.298 (1.953–2.704)	0.000	17.2%, 0.248
	Region					
	China	17	1817	2.327 (1.968–2.751)	0.000	20.6%, 0.213
	Other	1	74	1.880 (0.957–3.695)	0.067	–
	Cancer type					
	Estrogen-dependent carcinoma	2	198	2.656 (1.560–4.523)	0.000	0.0%, 0.457
	Urological carcinoma	3	321	1.952 (1.189–3.204)	0.008	51.4%, 0.128
	Glioma	4	430	2.315 (1.643–3.263)	0.000	0.0%, 0.534
	Digestive carcinoma	7	706	2.451 (1.862–3.227)	0.000	45.4%, 0.089
	Other	2	236	2.383 (1.449–3.920)	0.001	3.3%, 0.309
	Sample size					
	Sample < 100	7	528	2.017 (1.520–2.677)	0.000	10.4%, 0.350
	Sample > 100	11	1363	2.451 (2.009–2.990)	0.000	20.7%, 0.246

If I^2^ > 50%, the results were calculated by random model

*HR* hazard ratio, *CI* confidence interval

**Fig. 4:**
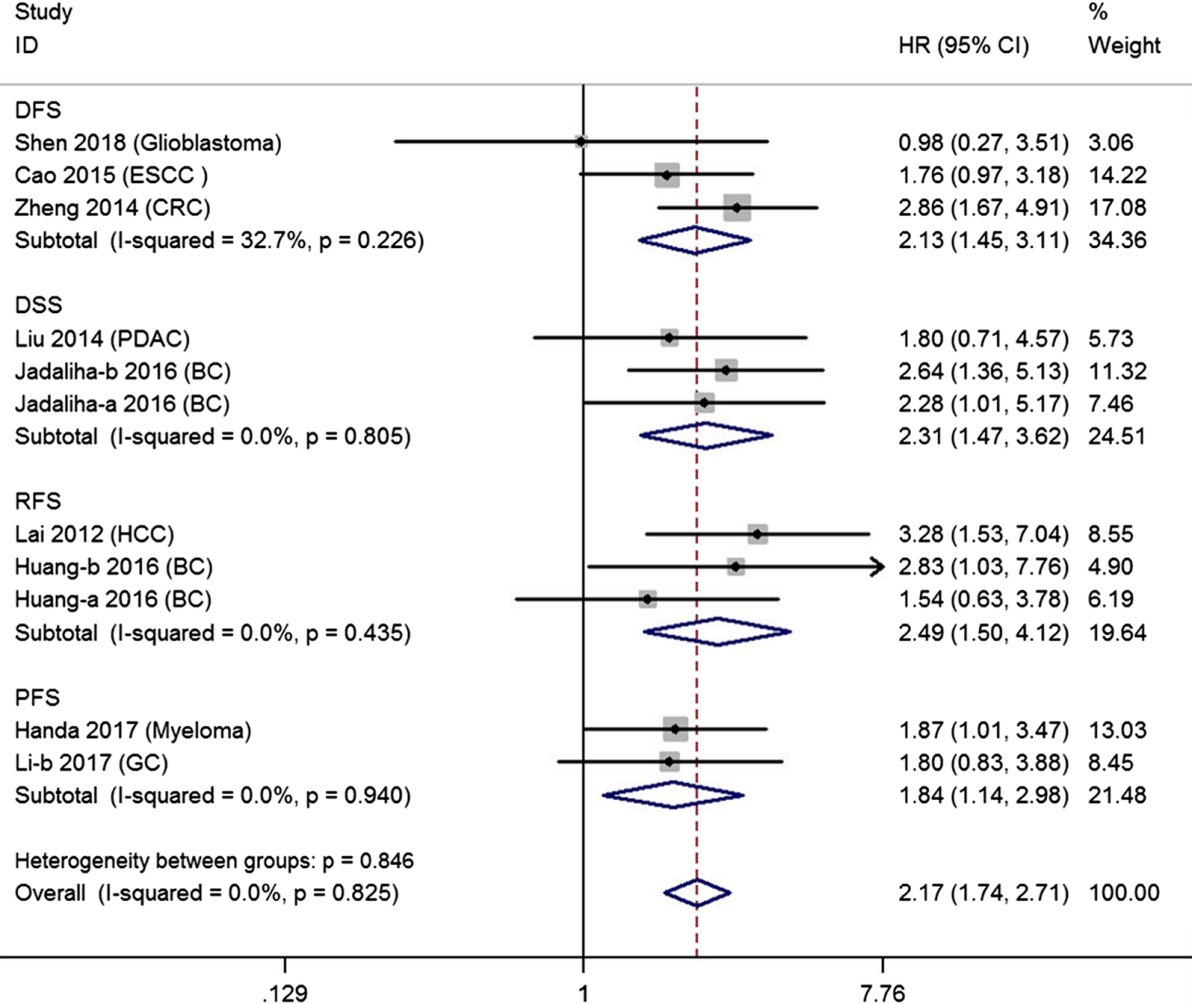
Forest plots of pooled HRs of DFS, RFS, DSS and PFS in multivariate model

Table 3 presented that patients with MALAT1 over-expression had shorter DFS (pooled HR = 2.036, 95% CI 1.240–3.342, P = 0.005), RFS (pooled HR = 2.491, 95% CI 1.505–4.123, P = 0.000), DSS (pooled HR = 2.098, 95% CI 1.372–3.211, P = 0.001) and PFS (pooled HR = 1.842, 95% CI 1.138–2.983, P = 0.013) than those with low MALAT1 expression in a fixed-effect model (shown in Table 3 and Fig. 4).

**Table 3:** Association between MALAT1 expression and RFS/DFS/DSS/PFS

Survival outcome	Survival analysis	Studies (n)	Patients (n)	HR (95% CI)	P	Heterogeneity (I^2^, *P* value)
RFS	Univariate	5	397	1.355 (0.751–2.445)	0.313	70.6%, 0.009
	Multivariate	3	263	2.491 (1.505–4.123)	0.000	0.0%, 0.435
DFS	Univariate	1	77	1.820 (1.018–3.255)	0.044	–
	Multivariate	3	329	2.036 (1.240–3.342)	0.005	32.7%, 0.226
DSS	Univariate	3	2037	1.791 (1.304–2.459)	0.000	4.5%, 0.351
	Multivariate	3	2037	2.098 (1.372–3.211)	0.001	0.0%, 0.578
PFS	Univariate	1	100	3.03 (1.578–5.820)	0.001	–
	Multivariate	2	175	1.842 (1.138–2.983)	0.013	0.0%, 0.940

If I^2^ > 50%, the results were calculated by random model

*DFS* disease-free survival, *RFS* recurrence-free survival, *DSS* disease-specific survival, *PFS* progression-free survival, *HR* hazard ratio, *CI* confidence interval

### Publication bias and sensitivity analysis

**Fig. 5:**
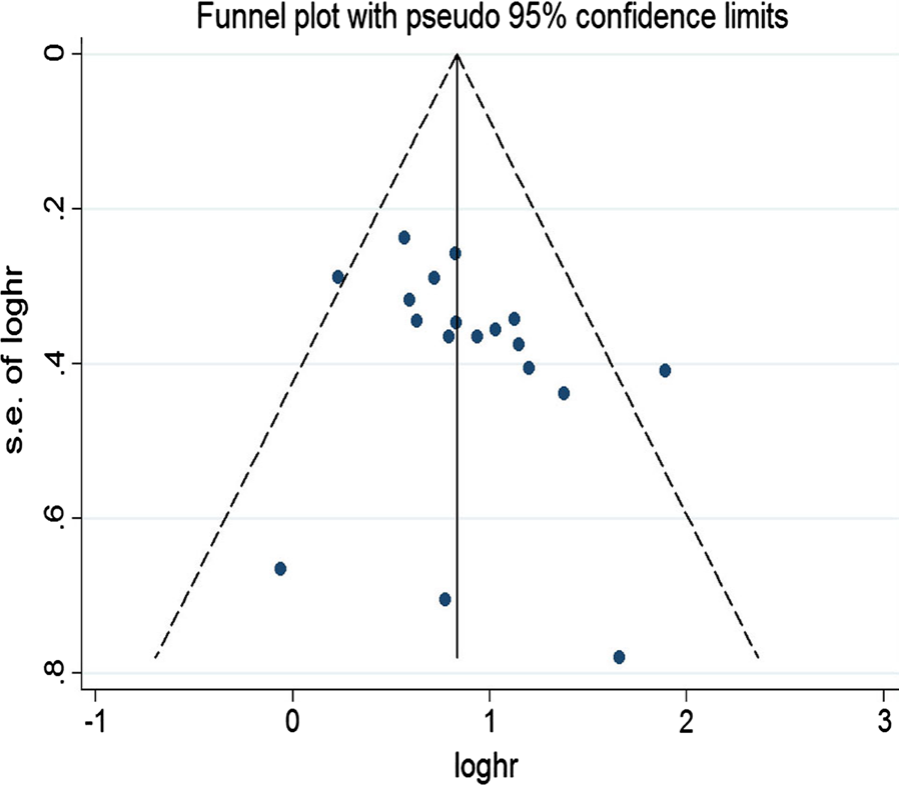
Funnel plots of OS in multivariate model

Egger’s test and Begg’s funnel plot were applied to examine publication bias. Egger’s test revealed absence of publication bias for OS in univariate (T = 1.47, P = 0.164) and multivariate (T = 1.55, P = 0.141) analyses. The symmetrical funnel plot of OS in multivariate model was presented in Fig. 5. The Egger’s tests and funnel plots of DFS, DSS and RFS also showed no publication bias. Furthermore, no significant publication bias was observed in clinicopathological parameters except for LNM (P = 0.041) and differentiation (P = 0.003). The studies of Droop et al. [36] significantly influenced the pooled results of OS and DFS according to sensitivity analysis, which indicated that the studies might explain the main source of heterogeneity across studies. Reanalyzed sensitivity analysis identified that the results of the study remained stability and robustness after getting rid of the studies of Droop et al.

### Carcinogenic mechanisms of MALAT1 among various cancers

To further explore the association of MALAT1 with the development of cancer, we summarized the effects of MALAT1 silencing on the malignant phenotype and its molecular mechanisms presented in Table 4. Additionally, MALAT1 possesses a variety of molecular functions including promotion of EMT [68], transcriptional dysregulation, pre-mRNA alternative splicing, ceRNA role [69], epigenetic alteration and transition of cell phenotype via different signaling pathways covering P13k/Akt [62], Wnt [18] and ERK/MAPK [70] pathways. Taken together, MALAT1 might promote carcinogenesis by exerting its molecular function to regulate the expression of related genes and activate the oncogenic signaling pathway [16, 46, 52, 64, 68–83].

**Table 4:** Research advances of MALAT1 in cancers (↑ represents promote; ↓ represents inhibit)

Cancer type	Expression effect	Transition of cell phenotype of MALAT1 silencing	Molecular mechanisms	References
Breast cancer	Up-regulated	G0/G1 phase cell cycle arrest; ↓ proliferation/migration/invasion	MALAT1 influenced cells progression by miR-129-5p; MALAT1 functions as a ceRNA of cdc42 in inducing EMT by binding miR-1 competitively	[13, 69]
Gallbladder cancer	Up-regulated	↓ Proliferation and metastasis	MALAT1 activates the ERK/MAPK pathway	[70]
Colorectal cancer	Up-regulated	↓ Proliferation/migration/invasion	Knockdown of AKAP-9 blocks MALAT1-mediated proliferation, migration and invasion; MALAT1 might bind to SFPQ, therefore releasing PTBP2 from the SFPQ/PTBP2 complex; SFPQ regulated the effect of MALAT1 on cell proliferation and migration	[79, 82]
Osteosarcoma	Up-regulated	↓ Invasion/proliferation/metastasis	MALAT1 knockdown inhabits PI3K/Akt pathways	[62]
Lung cancer	Up-regulated	↓ Migration/growth/invasion	MALAT1 regulates by TDP-43; MALAT1 silencing reduces the expression of CXCL5; the promoter methylation regulates MALAT1 expression; MALAT1 up-regulated the expression of miR-204 target gene SLUG through competitively ‘sponging’ miR-204	[16, 78, 81]
Pancreatic cancer	Up-regulated	G2/M cell cycle arrest; ↑ apoptosis; ↓ proliferation/metastasis/migration/invasion	MALAT1 promotes proliferation and metastasis by the stimulation of autophagy and inducing EMT	[68, 83]
Cervical cancer	Up-regulated	↓ Invasion/metastasis; ↑ apoptosis	MALAT1 regulates EMT by up-regulating transcriptional factor Snail	[46, 80]
Gastric cancer	Up-regulated	↓ Proliferation/migration/invasion/metastasis	MALAT1 regulates via increasing EGFL7 expression; MALAT1 regulates VE-cadherin/β-catenin complex, ERK/MMP and FAX/paxillin singling pathways involved in VM formation and angiogenesis; MALAT1 regulates miR-1297 to modulate HMGB2	[44, 64, 75]
Esophageal squamous cell carcinoma	Up-regulated	↓ Proliferation/migration/invasion/metastasis; ↑ apoptosis; G2/M phase cell cycle arrest	MALAT1 regulates the expression of β-catenin and Lin28 via Ezh2; Silencing of MALAT1 caused by miR-101 and miR-217; Knockdown of MALAT1 induces cell cycle arrest by activating the ATM-CHK2 pathway	[26, 58, 76, 77]
Hepatocellular carcinoma	Up-regulated	↓ Proliferation/invasion	MALAT1 regulates ZEB1 expression by sponging miR-143-3p	[14]
Renal cell carcinoma	Up-regulated	↓ Proliferation/invasion; ↑ apoptosis	Knockdown of MALAT1 significantly increases miR-205 expression in renal cancer cells; MALAT1 silencing elevated E-cadherin expression, whereas b-catenin expression reduced through Ezh2 to block EMT	[33]
Mantle cell lymphoma	Up-regulated	↓ Proliferation; ↑ apoptosis; S/G1 phase cell cycle arrest	MALAT1 represses the expression of PRC2-dependent target genes by ineracting with EZH2	[48]
Prostate cancer	Up-regulated	↓ Growth/invasion/migration; G0/G1 phase cell cycle arrest	MALAT1 increases oncogenic activities of EZH2	[73, 74]
Pancreatic ductal adenocarcinoma	Up-regulated	↓ Proliferation/migration/invasion/growth and cell cycle progression; ↑ apoptosis	MALAT1 acts as a ceRNA to regulate KRAS expression by sponging miR-217	[72]
Bladder cancer	Up-regulated	↓ Migration and metastasis	MALAT1 regulates TGF-b-induced EMT; MALAT1 promotes EMT by activating the Wnt/β-catenin signaling pathway	[17, 18]
Epithelial ovarian cancer	Up-regulated	↓ Proliferation/invasion/metastasis; ↑ apoptosis	MALAT1 induces EMT via PI3K/AKT pathway	[71]
Glioma	Down-regulated	↑ Proliferation/viability/progression	MALAT1 suppresses miR-155 expression and activates FBXW7 function	[52]

## Discussion

MALAT1 also known as NEAT2 (nuclear-enriched abundant transcript 2), is located in human chromosome 11q13. Unlike most of lncRNAs, MALAT1 is particularly abundant, highly conserved and ubiquitously expressed in multiple types of cancer. MALAT1 was originally discovered to predict metastasis and survival of non-small cell lung cancer [12]. Recently, increasing evidence provided that MALAT1 play a pivotal role in promoting proliferation, migration, metastasis and invasion of tumor cell. MALAT1, a multi-functional lncRNA, might involve in alternative splicing of pre-mRNA, transcriptional and post-transcriptional regulation via interacting with the relevant gene in carcinogenic pathways [84, 85]. Firstly, MALAT1, a novel transcript, may recruit a set of members of SR protein (serine/arginie riched protein) family, such as SRSF1, SRSF2, and SRSF3, and act as a “molecular sponge” to regulate SR protein activity, ultimately leading to alternative splicing of pre-mRNA [86]. Secondly, MALAT1 involvement in transcriptional dysregulation was supported by previous evidence, such as colocalization of serine-2 phosphorylated RNA polymerase II in nuclear speckle compartment, the interaction of unmethylated Pc2 with theTUG1, overlapping the histone H3K36me2 peaks and the recruitment of Sp1 on LTBP3 promoter. For example, MALAT1 could interact with unmethylated Pc2 in the nuclear speckles, and regulate the localization of the Pc2, together with theTUG1, whereas methylated Pc2 generally exists in other nuclear bodies [87]. MALAT1 also inclined to cooperate with the 3′ end of the gene body, overlapping the histone H3K36me2 peaks, a biomarker of active transcriptional elongation [10]. Thirdly, the mechanisms of the post-transcriptional regulation of MALAT1 mainly contains alternative splicing, protein activities and ceRNAs. For example, MALAT1, as a ceRNA, could reciprocally interacts with microRNAs (miR-205, miR-1297, miR-217 and miR-155), ultimately contributing to cell phenotypic changes such as invasiveness, metastasis, proliferation, migration and apoptosis [52, 72, 77]. Besides, MALAT1 might influence carcinogenesis of cancers by activating Wnt/β-catenin, ERK/MAPK and PI3K/AKT pathways, which simultaneous activation of the oncogenic pathways might bring out highly carcinogenic effects [88]. For example, knockdown of MALAT1 could induce the EMT by regulating transcriptional factor snail and activating the PI3K/AKT and Wnt pathways [31, 33, 80]. Furthermore, upregulated MALAT1 could promote EMT-mediated cell migration and metastasis of various malignant cancers since its inhibition impairs the effect of TGF-β-induced EMT by suppressor of zeste 12 (suz12) [17]. Hence, emerging studies have implied that MALAT1 could serve as a potential prognostic biomarker for cancer patients on the basis of the complicated mechanisms of MALAT1 among multiple types of cancer.

Previous published articles reported that lncRNAs including MALAT1 are effective predictors of survival outcomes [89, 90]. However, MALAT1 on the influence of prognostic outcome is still controversial. Therefore, we combined the published studies to evaluate the prognostic and clinical value of MALAT1 among different types of cancer. The meta-analysis is firstly to investigate the relationship between MALAT1 expression and prognosis of glioma as well as estrogen-dependent cancer by pooling eligible studies in multivariate model, which is different from previous meta-analyses. The pooled results of multivariate model may be closer to revealing the authentic relationship of MALAT1 expression with different types of cancers since the adjusted confounding factors involved in several clinical variables as confounders including LNM, differentiation, distant metastasis and other factors. This study also firstly analyzed the association of MALAT1 with tumour differentiation, distant metastasis, TNM stage and tumour size.

The results of the study identified that patients with high expression of MALAT1 have a poor OS in univariate and multivariate models. Moreover, over-expression of MALAT1 may be an unfavorable biomarker of DFS, RFS, DSS and PFS. The results also indicated that the adverse prognostic effect of MALAT1 over-expression was obtained in different types of cancer (estrogen-dependent cancer: pooled HR = 2.656; urological cancer: pooled HR = 1.952; glioma: pooled HR = 2.315; digestive cancer: pooled HR = 2.451). The results of the study are consistent with most of the original studies, which suggest that MALAT1 may be associated with poor prognosis in malignant cancers [61–63]. However, the sensitivity analysis identified the studies of Droop et al. [36], which influenced the stability of the pooled results. The possible reasons were as follows. First, the heterogeneity across studies may be attributed to the difference of genetic background since the subjects of the study were from Germany. Second, Droop et al.’ the study involved two types of bladder cancer, including non-muscle-invasive tumours (NMIBC) and muscle-invasive bladder cancer (MIBC). NMIBC is essentially different from MIBC in tumor biology. Finally, the study is a small sample study. These potential confounders might account for the heterogeneity across studies. In addition, we need to explain that publication bias of LNM and differentiation may be due to small sample studies, which are susceptible to publication bias. Therefore, based on the above evidence, the abnormal regulation and prognostic utility of MALAT1 across multiple types of tumors suggests that MALAT may be a candidate biomarker for applying to therapeutic targets for clinical practice.

There are several limitations in the study. First, the cut-off values of high and low MALAT1 expression were different across studies. Second, the heterogeneity among studies may be due to different qRT-PCR primer sets. Third, significant heterogeneity may also be caused by confounding factors, such as cancer type, ethnicity, and other confounders. Fourth, several original studies did not provide complete data. Finally, the study might present “small-study effects” [91, 92]. For example, the last meta-analysis of HOXA11-AS demonstrated that small sample size studies with lacking of statistical power could obtain higher effect size compared with large sample studies [93]. Therefore, larger-scale studies are authorized to verify these results of the study.

## Conclusions

In conclusion, the study revealed that over-expression MALAT1 might be an adverse biomarker for prognostic outcome, lymph node metastasis, distant metastasis, tumour size and TNM stage for cancer patients. MALAT1 might play a pivotal role in the tumorigenesis of multiple types of cancers. However, more high-quality larger-scale studies across ethnicities are warranted to explore the prognostic value and carcinogenic function of MALAT1 before it is applied to the treatment and management of cancer.

## Additional files

**Additional file 1: Table S1.** The characteristics of the included studies in the meta-analysis.

**Additional file 2: Table S2.** Characteristics of studies for survival outcomes included in the meta-analysis.

**Additional file 3: Table S3.** Association between MALAT1 expression and clinicopathological features of cancers.

## Abbreviations

LncRNAs: long noncoding RNAs
MALAT1: the metastasis-associated lung adenocarcinoma transcript 1
qRT-PCR: quantitative real-time polymerase chain reaction
ISH: in situ hybridization
HWE: hardy–weinberg equilibrium
HR: hazard ratio
OS: overall survival
DFS: disease-free survival
RFS: recurrence-free survival
DSS: disease-specific survival
PFS: progression-free survival
GC: gastric cancer
GBC: gallbladder cancer
EC: esophageal cancer
PDAC: pancreatic duct adenocarcinoma
ESCC: esophageal squamous cell carcinoma
HCC: hepatocellular carcinoma
CRC: colorectal cancer
GBM: glioblastoma multiforme
EOC: epithelial ovarian cancer
BC: breast cancer
UC: urothelial carcinoma
ccRCC: clear cell renal cell carcinoma
SR: serine/arginie-rich
EMT: epithelial–mesenchymal transition
LNM: lymph node metastasis
ceRNA: competitive endogenous RNA
