# Supplementary material for: Clinicopathological and prognostic significance of long noncoding RNA MALAT1 in human cancers: a review and meta-analysis
Source: Cancer Cell Int. 2018 Aug 6;18:109. doi: 10.1186/s12935-018-0606-z (PMC6080354; doi:10.1186/s12935-018-0606-z)
Supplement: Supplementary file 1 — Additional file 1: Table S1. The characteristics of the included studies in the meta-analysis. [file 12935_2018_606_MOESM1_ESM.docx]

**Table S1** The characteristics of the included studies in the meta-analysis

| **First author** | **Year** | **Country** | **Type of cancers** | **Cut-off value** | **Follow-up(months)** | **Detection method** | **Simple size** | **survival outcome**  **(analysis model)** | **Clinicopathological**  **parameters included in the meta-analysis** |
| --- | --- | --- | --- | --- | --- | --- | --- | --- | --- |
| Sun [[51](#_ENREF_51)] | 2018 | China | GBC | ROC | 60 | qRT-PCR | 102 | OS (M) | N/A |
| Shen [[28](#_ENREF_28)] | 2018 | China | Glioblastoma | N/A | 24 | qRT-PCR | 106 | OS,DFS (M) | N/A |
| Xiong [[57](#_ENREF_57)] | 2018 | China | CRC | N/A | 60 | qRT-PCR | 58 | N/A | Gender, LNM, DM, Differentiation |
| Handa [[35](#_ENREF_35)] | 2017 | Japan | Myeloma | Median | 21(median) | qRT-PCR | 74 | OS,PFS (M) | N/A |
| Sonohara [[22](#_ENREF_22)] | 2017 | Japan | HCC | Median | 120 | qRT-PCR | 240 | OS,RFS (U) | N/A |
| Li [[32](#_ENREF_32)] | 2017 | China | Bladder cancer | Mean | 61.8(median) | qRT-PCR | 120 | OS (U,M) | Age, Gender, Tumor size, LNM, Differentiation |
| Li [[44](#_ENREF_44)] | 2017 | China | GC | N/A | 50(total) | ISH | 150 | OS,PFS (U,M) | Age, Gender, LNM, DM, Differentiation, TNM stage |
| Droop [[36](#_ENREF_36)] | 2017 | Germany | UC | Median | 22.7(median) | qRT-PCR | 106 | OS,DFS (U,M) | N/A |
| Chen [[41](#_ENREF_41)] | 2017 | China | GBM | ROC | 30 | qRT-PCR | 140 | OS (U,M) | N/A |
| Zhang [[54](#_ENREF_54)] | 2017 | China | GC | Median | N/A | qRT-PCR | 60 | N/A | Age, Gender, Tumor size, LNM, Differentiation, TNM stage |
| Li[[64](#_ENREF_64)] | 2017 | China | GC | Median | <70 | qRT-PCR | 78 | N/A | Age, Gender, LNM, DM, Differentiation, TNM stage |
| Wang [[56](#_ENREF_56)] | 2017 | China | Osteosarcoma | N/A | 60 | qRT-PCR | 45 | N/A | Age, Gender, Tumor size, DM |
| Zuo [[13](#_ENREF_13)] | 2017 | China | BC | Median | 60 | qRT-PCR | 43 | N/A | Age, Tumor size, LNM, DM |
| Zhang [[53](#_ENREF_53)] | 2017 | China | NSCLC | N/A | N/A | qRT-PCR | 77 | N/A | Age, Gender, Tumor size, LNM, Differentiation |
| Jen [[55](#_ENREF_55)] | 2017 | China | LC | N/A | >150 | qRT-PCR | 124 | N/A | LNM, DM, TNM stage |
| Wu[[66](#_ENREF_66)] | 2017 | China | OC | Mean | N/A | qRT-PCR | 42 | N/A | Age, Tumor size, LNM, Differentiation |
| Wang [[48](#_ENREF_48)] | 2016 | China | MCL | Median | <60 | qRT-PCR | 40 | OS (U) | Age, Gender |
| Duan [[38](#_ENREF_38)] | 2016 | China | Bladder cancer | Median | 76 | qRT-PCR | 95 | RFS,RFS (U) | N/A |
| Cao [[52](#_ENREF_52)] | 2016 | China | Glioma | Median | <60 | qRT-PCR | 66 | OS (M) | N/A |
| Huang [[50](#_ENREF_50)] | 2016 | China | EC | Mean | 60 | qRT-PCR | 133 | OS (M) | Age, Gender, DM, Differentiation |
| Gao [[42](#_ENREF_42)] | 2016 | China | Osteosarcoma | Median | 70 | qRT-PCR | 162 | OS (U,M) | Age, Gender, Tumor size, DM |
| Xia [[45](#_ENREF_45)] | 2016 | China | GC | ROC | 50 | qRT-PCR | 72 | OS (U,M) | N/A |
| Chen [[29](#_ENREF_29)] | 2016 | China | EOC | Median | 50 | qRT-PCR | 94 | OS (U,M) | N/A |
| Jadaliha [[39](#_ENREF_39)] | 2016 | USA | BC | N/A | N/A | qRT-PCR | 1992 | DSS (U,M) | N/A |
| Huang [[49](#_ENREF_49)] | 2016 | China | BC | 0.75 | 65 | qRT-PCR | 365 | RFS (U,M) | Age, Tumor Size, LNM |
| Yao [[26](#_ENREF_26)] | 2016 | China | ESCC | N/A | 36 | qRT-PCR | 137 | N/A | Age, Gender, LNM, Differentiation |
| Li [[59](#_ENREF_59)] | 2016 | China | CRC | Median | <30 | qRT-PCR | 30 | N/A | Age, Gender, TNM stage |
| Qiu [[25](#_ENREF_25)] | 2016 | China | HCC | Median | 53.6(median) | qRT-PCR | 120 | N/A | Age, Gender, Tumor size, Differentiation, TNM stage |
| Miao [[67](#_ENREF_67)] | 2016 | China | BC | Median | <100 | qRT-PCR | 78 | N/A | Age, Tumor Size, LNM, Differentiation |
| Wang [[65](#_ENREF_65)] | 2016 | China | GBC | Median | 40 | qRT-PCR | 30 | N/A | Age, Gender, Tumor size, LNM, Differentiation, |
| Luo [[63](#_ENREF_63)] | 2016 | China | HCC | N/A | 30 | qRT-PCR | 32 | N/A | Age, Gender, Tumor size, TNM stage |
| Jin [[60](#_ENREF_60)] | 2016 | China | NPC | SI>=6(high) | <60 | ISH | 131 | N/A | Age, Gender, LNM, DM, TNM stage |
| Yang [[46](#_ENREF_46)] | 2015 | china | CC | Median | 30(median) | qRT-PCR | 104 | OS (U,M) | Age, Tumor size, LNM, Differentiation |
| Ma [[27](#_ENREF_27)] | 2015 | China | Glioma | Median | 60 | qRT-PCR | 118 | OS (U,M) | Age, Gender, Tumor size |
| Pang [[24](#_ENREF_24)] | 2015 | China | PDAC | Median | 60 | qRT-PCR | 126 | OS (U,M) | Age, Gender, Tumor size LNM, DM, Differentiation |
| Zhang [[34](#_ENREF_34)] | 2015 | China | ccRCC | Mean | 60 | qRT-PCR | 106 | OS (U,M) | Age, Gender, LNM, DM, Differentiation |
| Cao [[37](#_ENREF_37)] | 2015 | China | ESCC | N/A | 37(median) | qRT-PCR | 77 | OS (U) | Age, Gender, LNM, Differentiation, TNM stage |
| Hirata [[33](#_ENREF_33)] | 2015 | Japan | ccRCC | Median | 60 | qRT-PCR | 50 | N/A | Age, Gender, LNM, DM |
| Dong [[62](#_ENREF_62)] | 2015 | China | Osteosarcoma | Mean | 60 | qRT-PCR | 19 | N/A | Gender, LNM, DM, |
| Jin[[61](#_ENREF_61)] | 2016 | China | BC | Median | N/A | qRT-PCR | 139 | N/A | Age, Tumor size, DM, TNM stage |
| Xu[[31](#_ENREF_31)] | 2015 | China | BC | Median | <60 | qRT-PCR | 135 | N/A | Age, LNM, Differentiation |
| Hu[[58](#_ENREF_58)] | 2015 | China | ESCC | N/A | N/A | qRT-PCR | 54 | N/A | Age, gender, LNM |
| Okugawa [[20](#_ENREF_20)] | 2014 | Japan | GC | ROC | 60 | qRT-PCR | 150 | OS (U) | Age, Gender, Tumor size, LNM |
| Zheng [[47](#_ENREF_47)] | 2014 | China | CRC | Median | 56.2(median) | qRT-PCR | 146 | OS, DFS (M) | Age, Gender, LNM, Differentiation, TNM stage |
| Liu [[23](#_ENREF_23)] | 2014 | China | PDAC | ROC | 47 | qRT-PCR | 45 | DSS (U,M) | Age, Gender, Tumor size, LNM, DM, Differentiation |
| Fan [[17](#_ENREF_17)] | 2014 | China | Bladder cancer | N/A | N/A | qRT-PCR | 95 | OS (M) | Age, Gender, LNM, DM, Differentiation |
| Lai [[43](#_ENREF_43)] | 2012 | China | HCC | Mean | 18.6(median) | qRT-PCR | 60 | RFS (U,M) | Age, Gender, Tumor size, Differentiation |
| Schmidt [[40](#_ENREF_40)] | 2011 | Germany | NSCLC | N/A | 38.2(median) | ISH | 352 | OS (U) | N/A |

NPC Nasopharyngeal carcinoma, GBC Gallbladder cancer, MCL Mantle cell lymphoma, UC Urothelial carcinoma, EC Esophageal cancer, CRC Colorectal cancer, CC Cervical cancer, GBM Glioblastoma multiforme, PDAC Pancreatic duct adenocarcinoma, NMIBC Non-muscle invasive bladder cancer, MIBC Muscle-invasive bladder cancer, GC Gastric cancer, ESCC Esophageal squamous cell carcinoma, HCC Hepatocellular carcinoma, EOC Epithelial ovarian cancer, BC Breast cancer, ccRCC clear cell renal cell carcinoma, Non-small cell lung cancer NSCLC, DFS Disease-free survival, RFS Recurrence-free survival, DSS Disease-specific survival, PFS Progression-free survival, OS Overall survival, M Multivariate, U Univariate, N/A not available.
