# Supplementary material for: Clinicopathological and prognostic significance of long noncoding RNA MALAT1 in human cancers: a review and meta-analysis
Source: Cancer Cell Int. 2018 Aug 6;18:109. doi: 10.1186/s12935-018-0606-z (PMC6080354; doi:10.1186/s12935-018-0606-z)
Supplement: Supplementary file 2 — Additional file 2: Table S2. Characteristics of studies for survival outcomes included in the meta-analysis. [file 12935_2018_606_MOESM2_ESM.docx]

**Table S****2** Characteristics of studies for survival outcomes included in the meta-analysis

| **First author** | **Year** | **Country** | **Type of cancers** | **Cut-off value** | | **Follow-up(months)** | **Detection method** | **Simple Size** | **Survival outcomes** | **Analysis**  **model** | **HR** | **LCI** | **UCI** |
| --- | --- | --- | --- | --- | --- | --- | --- | --- | --- | --- | --- | --- | --- |
| Sun[[51](#_ENREF_51)] | 2018 | China | GBC | ROC | 60 | | qRT-PCR | 102 | OS | M | 2.29 | 1.15 | 4.56 |
| Shen[[28](#_ENREF_28)] | 2018 | China | Glioblastoma | N/A | 24 | | qRT-PCR | 106 | OS | M | 0.94 | 0.23 | 3.21 |
| Shen[[28](#_ENREF_28)] | 2018 | China | Glioblastoma | N/A | 24 | | qRT-PCR | 106 | DFS | M | 0.98 | 0.24 | 3.16 |
| Handa[[35](#_ENREF_35)] | 2017 | Japan | Myeloma | Median | 21(median) | | qRT-PCR | 74 | OS | M | 1.88 | 0.95 | 3.72 |
| Handa[[35](#_ENREF_35)] | 2017 | Japan | Myeloma | Median | 21(median) | | qRT-PCR | 75 | PFS | M | 1.87 | 1.00 | 3.49 |
| Sonohara[[22](#_ENREF_22)] | 2017 | Japan | HCC | Median | 120 | | qRT-PCR | 158 | OS | U | 0.58 | 0.36 | 0.95 |
| Sonohara[[22](#_ENREF_22)] | 2017 | Japan | HCC | Median | 120 | | qRT-PCR | 81 | RFS | U | 0.78 | 0.48 | 1.30 |
| Li[[32](#_ENREF_32)] | 2017 | China | Bladder cancer | Mean | 61.8(median) | | qRT-PCR | 120 | OS | U | 3.02 | 1.80 | 5.29 |
|  |  |  |  |  |  | |  |  | OS | M | 2.06 | 1.24 | 3.88 |
| Li-a[[44](#_ENREF_44)] | 2017 | China | GC | N/A | 50 | | ISH | 50 | OS | U | 4.39 | 1.33 | 14.49 |
|  |  |  |  |  |  | |  |  | OS | M | 5.26 | 1.12 | 24.61 |
| Li-b[[44](#_ENREF_44)] | 2017 | China | GC | N/A | 50 | | ISH | 100 | PFS | U | 3.03 | 1.57 | 5.87 |
|  |  |  |  |  |  | |  |  | PFS | M | 1.80 | 0.83 | 3.92 |
| Droop[[36](#_ENREF_36)] | 2017 | Germany | UC | Median | 22.7(median) | | qRT-PCR | 106 | OS | U | 0.55 | 0.35 | 0.85 |
|  |  |  |  |  |  | |  |  | OS | M | 0.63 | 0.40 | 0.98 |
| Droop[[36](#_ENREF_36)] | 2017 | Germany | UC | Median | 22.7(median) | | qRT-PCR | 106 | DFS | U | 0.59 | 0.36 | 0.78 |
|  |  |  |  |  |  | |  |  | DFS | M | 0.77 | 0.46 | 1.28 |
| Chen[[41](#_ENREF_41)] | 2017 | China | GBM | ROC | 30 | | qRT-PCR | 140 | OS | U | 2.32 | 1.20 | 4.88 |
|  |  |  |  |  |  | |  |  | OS | M | 2.55 | 1.22 | 5.20 |
| Wang[[48](#_ENREF_48)] | 2016 | China | MCL | Median | <60 | | qRT-PCR | 40 | OS | U | 3.01 | 1.01 | 8.30 |
| Duan-a[[38](#_ENREF_38)] | 2016 | China | NMIBC | Median | 76 | | qRT-PCR | 59 | RFS | U | 0.61 | 0.28 | 1.32 |
| Duan-b[[38](#_ENREF_38)] | 2016 | China | MIBC | Median | 76 | | qRT-PCR | 36 | RFS | U | 1.83 | 0.79 | 4.23 |
| Cao[[52](#_ENREF_52)] | 2016 | China | Glioma | Median | <60 | | qRT-PCR | 66 | OS | M | 2.80 | 1.16 | 4.74 |
| Huang[[50](#_ENREF_50)] | 2016 | China | EC | Mean | 60 | | qRT-PCR | 133 | OS | M | 6.64 | 2.95 | 14.95 |
| Gao[[42](#_ENREF_42)] | 2016 | China | Osteosarcoma | Median | 70 | | qRT-PCR | 162 | OS | U | 2.80 | 1.76 | 7.84 |
|  |  |  |  |  |  | |  |  | OS | M | 3.16 | 1.56 | 6.88 |
| Xia[[45](#_ENREF_45)] | 2016 | China | GC | ROC | 50 | | qRT-PCR | 72 | OS | U | 3.26 | 0.22 | 5.10 |
|  |  |  |  |  |  | |  |  | OS | M | 2.17 | 0.27 | 4.32 |
| Chen[[29](#_ENREF_29)] | 2016 | China | EOC | Median | 50 | | qRT-PCR | 94 | OS | U | 2.39 | 1.06 | 4.67 |
|  |  |  |  |  |  | |  |  | OS | M | 3.32 | 1.14 | 5.68 |
| Jadaliha-a[[39](#_ENREF_39)] | 2016 | USA | BC | N/A | N/A | | qRT-PCR | 1992 | DSS(HER2+) | U | 2.32 | 1.02 | 5.31 |
|  |  |  |  |  |  | |  |  | DSS(HER2+) | M | 2.28 | 1.00 | 5.22 |
| Jadaliha-b[[39](#_ENREF_39)] | 2016 | USA | BC | N/A | N/A | | qRT-PCR | 1992 | DSS(basal-like) | U | 2.46 | 1.27 | 4.78 |
|  |  |  |  |  |  | |  |  | DSS(basal-like) | M | 2.64 | 1.35 | 5.16 |
| Huang-a[[49](#_ENREF_49)] | 2016 | China | BC | 0.75 | 65 | | qRT-PCR | 118 | RFS(ER+) | M | 1.54 | 0.62 | 3.80 |
| Huang-b[[49](#_ENREF_49)] | 2016 | China | BC | 0.75 | 65 | | qRT-PCR | 85 | RFS(ER-) | M | 2.83 | 1.02 | 7.83 |
| Huang-c[[49](#_ENREF_49)] | 2016 | China | BC | 0.75 | 65 | | qRT-PCR | 161 | RFS(ER+) | U | 2.56 | 1.04 | 6.00 |
| Yang[[46](#_ENREF_46)] | 2015 | china | CC | Median | 30(median) | | qRT-PCR | 104 | OS | U | 4.23 | 2.20 | 8.13 |
|  |  |  |  |  |  | |  |  | OS | M | 2.21 | 1.07 | 4.57 |
| Ma[[27](#_ENREF_27)] | 2015 | China | Glioma | Median | 60 | | qRT-PCR | 118 | OS | U | 3.00 | 1.82 | 4.94 |
|  |  |  |  |  |  | |  |  | OS | M | 2.29 | 1.37 | 3.81 |
| Pang[[24](#_ENREF_24)] | 2015 | China | PDAC | Median | 60 | | qRT-PCR | 126 | OS | U | 2.44 | 1.58 | 3.77 |
|  |  |  |  |  |  | |  |  | OS | M | 1.76 | 1.10 | 2.82 |
| Zhang[[34](#_ENREF_34)] | 2015 | China | ccRCC | Mean | 60 | | qRT-PCR | 106 | OS | U | 3.52 | 2.05 | 8.93 |
|  |  |  |  |  |  | |  |  | OS | M | 3.09 | 1.81 | 7.03 |
| Cao[[37](#_ENREF_37)] | 2015 | China | ESCC | N/A | 37(median) | | qRT-PCR | 77 | OS | U | 1.89 | 1.02 | 3.50 |
|  |  |  |  |  |  | |  |  | OS | M | 1.81 | 0.97 | 3.41 |
| Cao[[37](#_ENREF_37)] | 2015 | China | ESCC | N/A | 37(median) | | qRT-PCR | 77 | DFS | U | 1.82 | 1.01 | 3.27 |
|  |  |  |  |  |  | |  |  | DFS | M | 1.76 | 0.97 | 3.21 |
| Okugawa[[20](#_ENREF_20)] | 2014 | Japan | GC | ROC | 60 | | qRT-PCR | 150 | OS | U | 1.54 | 0.92 | 2.58 |
| Zheng[[47](#_ENREF_47)] | 2014 | China | CRC | Median | 56.2(median) | | qRT-PCR | 146 | OS | M | 3.97 | 1.67 | 9.46 |
|  |  |  |  |  |  | |  |  | DFS | M | 2.86 | 1.66 | 4.94 |
| Liu [[23](#_ENREF_23)] | 2014 | China | PDAC | ROC | 47 | | qRT-PCR | 45 | DSS | U | 1.51 | 1.03 | 2.21 |
|  |  |  |  |  |  | |  |  | DSS | M | 1.80 | 1.18 | 7.75 |
| Fan[[17](#_ENREF_17)] | 2014 | China | Bladder cancer | N/A | N/A | | qRT-PCR | 95 | OS | M | 1.26 | 0.68 | 2.13 |
| Lai[[43](#_ENREF_43)] | 2012 | China | HCC | Mean | 18.6(median) | | qRT-PCR | 60 | RFS | U | 2.44 | 1.18 | 5.02 |
|  |  |  |  |  |  | |  |  | RFS | M | 3.28 | 1.52 | 7.09 |
| Schmidt[[40](#_ENREF_40)] | 2011 | Germany | NSCLC | N/A | 38.2(median) | | ISH | 352 | OS | U | 1.78 | 1.08 | 2.92 |

GBC Gallbladder cancer, MCL Mantle cell lymphoma, UC Urothelial carcinoma, EC Esophageal cancer, CRC Colorectal cancer, CC Cervical cancer, GBM Glioblastoma multiforme, PDAC Pancreatic duct adenocarcinoma, NMIBC Non-muscle invasive bladder cancer, MIBC Muscle-invasive bladder cancer, GC Gastric cancer, ESCC Esophageal squamous cell carcinoma, HCC Hepatocellular carcinoma, EOC Epithelial ovarian cancer, BC Breast cancer, ccRCC clear cell renal cell carcinoma, Non-small cell lung cancer NSCLC,M Multivariate, U Univariate, DFS Disease-free survival, RFS recurrence-free survival, DSS disease-specific survival, PFS progression-free survival, OS overall survival.
