# Supplementary material for: Clinicopathological and prognostic significance of long noncoding RNA MALAT1 in human cancers: a review and meta-analysis
Source: Cancer Cell Int. 2018 Aug 6;18:109. doi: 10.1186/s12935-018-0606-z (PMC6080354; doi:10.1186/s12935-018-0606-z)
Supplement: Supplementary file 3 — Additional file 3: Table S3. Association between MALAT1 expression and clinicopathological features of cancers. [file 12935_2018_606_MOESM3_ESM.docx]

**Table S3** Association between MALAT1 expression and clinic-pathological features of cancers

| **Characteristics** | **Age (elderly vs. nonelderly)** | | | | **Gender (female vs. male )** | | | | **Tumor size (cm) (large size vs. small size)** | | | | **Lymph node metastasis (positive vs. negative)** | | | | **Distant metastasis (presence vs. absence)** | | | | **Differentiation (poor vs. well, moderate)** | | | | **TNM Stage (III +IV vs. I + II)** | | | |
| --- | --- | --- | --- | --- | --- | --- | --- | --- | --- | --- | --- | --- | --- | --- | --- | --- | --- | --- | --- | --- | --- | --- | --- | --- | --- | --- | --- | --- |
| Study ID | a1 | a0 | b1 | b0 | a1 | a0 | b1 | b0 | a1 | a0 | b1 | b0 | a1 | a0 | b1 | b0 | a1 | a0 | b1 | b0 | a1 | a0 | b1 | b0 | a1 | a0 | b1 | b0 |
| Cao [[37](#_ENREF_37)] | 14 | 31 | 11 | 21 | 10 | 35 | 10 | 22 | - | - | - | - | 16 | 29 | 11 | 21 | - | - | - | - | 18 | 27 | 12 | 20 | 15 | 30 | 7 | 25 |
| Dong [[62](#_ENREF_62)] | - | - | - | - | 5 | 9 | 2 | 3 | - | - | - | - | 14 | 1 | 4 | 1 | 11 | 4 | 1 | 4 | - | - | - | - | - | - | - | - |
| Fan [[17](#_ENREF_17)] | 31 | 14 | 38 | 12 | 7 | 38 | 16 | 34 | - | - | - | - | 20 | 25 | 13 | 37 | 16 | 29 | 10 | 40 | 8 | 37 | 5 | 45 | - | - | - | - |
| Gao [[42](#_ENREF_42)] | 20 | 60 | 28 | 54 | 47 | 33 | 42 | 40 | 20 | 60 | 26 | 56 | - | - | - | - | 31 | 49 | 13 | 69 | - | - | - | - | - | - | - | - |
| Hirata [[33](#_ENREF_33)] | 15 | 10 | 16 | 9 | 9 | 16 | 7 | 18 | - | - | - | - | 4 | 21 | 0 | 25 | 3 | 22 | 0 | 25 | - | - | - | - | - | - | - | - |
| Hu[[58](#_ENREF_58)] | 12 | 13 | 14 | 15 | 11 | 14 | 11 | 18 | - | - | - | - | 16 | 9 | 8 | 21 | - | - | - | - | - | - | - | - | - | - | - | - |
| Huang [[49](#_ENREF_49)] | 53 | 49 | 53 | 49 | - | - | - | - | 61 | 41 | 69 | 33 | 49 | 53 | 46 | 56 | - | - | - | - | - | - | - | - | - | - | - | - |
| Huang [[50](#_ENREF_50)] | 54 | 45 | 16 | 18 | 41 | 58 | 19 | 15 | - | - | - | - | - | - | - | - | 52 | 47 | 10 | 24 | 56 | 43 | 11 | 23 | - | - | - | - |
| Jen[[55](#_ENREF_55)] | - | - | - | - | - | - | - | - | - | - | - | - | 48 | 59 | 2 | 12 | 7 | 102 | 0 | 14 | - | - | - | - | 49 | 60 | 2 | 12 |
| Jin [[60](#_ENREF_60)] | 31 | 35 | 36 | 29 | 32 | 34 | 39 | 26 | - | - | - | - | 49 | 29 | 21 | 32 | 48 | 34 | 19 | 30 | - | - | - | - | 44 | 36 | 17 | 34 |
| Jin[[61](#_ENREF_61)] | 40 | 29 | 45 | 25 | - | - | - | - | 43 | 26 | 47 | 23 | - | - | - | - | 17 | 52 | 31 | 39 | - | - | - | - | 26 | 43 | 39 | 31 |
| Lai [[43](#_ENREF_43)] | 13 | 20 | 13 | 14 | 3 | 30 | 2 | 25 | 18 | 15 | 16 | 11 | - | - | - | - | - | - | - | - | 10 | 23 | 8 | 19 | - | - | - | - |
| Li[[32](#_ENREF_32)] | 24 | 40 | 22 | 34 | 36 | 28 | 30 | 26 | 29 | 35 | 28 | 28 | 21 | 43 | 7 | 49 | - | - | - | - | 18 | 46 | 27 | 29 | - | - | - | - |
| Li[[44](#_ENREF_44)] | 63 | 42 | 23 | 22 | 38 | 67 | 18 | 27 | - | - | - | - | 93 | 12 | 25 | 20 | 41 | 64 | 9 | 36 | 51 | 54 | 29 | 16 | 86 | 19 | 18 | 27 |
| Li [[59](#_ENREF_59)] | 5 | 10 | 7 | 8 | 7 | 8 | 8 | 7 | - | - | - | - | - | - | - | - | - | - | - | - | - | - | - | - | 4 | 11 | 6 | 9 |
| Li[[64](#_ENREF_64)] | 15 | 23 | 18 | 22 | 20 | 18 | 16 | 24 | - | - | - | - | 22 | 18 | 13 | 27 | 10 | 28 | 18 | 22 | 16 | 22 | 19 | 21 | 25 | 13 | 17 | 23 |
| Liu [[23](#_ENREF_23)] | 12 | 14 | 12 | 7 | 11 | 15 | 8 | 11 | 21 | 5 | 9 | 10 | 15 | 11 | 8 | 11 | 6 | 20 | 1 | 18 | 7 | 19 | 2 | 17 | - | - | - | - |
| Luo [[63](#_ENREF_63)] | 8 | 7 | 8 | 9 | 3 | 12 | 4 | 13 | 12 | 3 | 5 | 12 | - | - | - | - | - | - | - | - | - | - | - | - | 14 | 1 | 8 | 9 |
| Ma [[27](#_ENREF_27)] | 34 | 25 | 39 | 20 | 31 | 28 | 24 | 35 | 43 | 16 | 29 | 30 | - | - | - | - | - | - | - | - | - | - | - | - | - | - | - | - |
| Miao [[67](#_ENREF_67)] | 16 | 23 | 20 | 19 | - | - | - | - | 24 | 15 | 22 | 17 | 28 | 11 | 19 | 20 | - | - | - | - | 19 | 20 | 16 | 23 | - | - | - | - |
| Okugawa [[20](#_ENREF_20)] | 47 | 41 | 32 | 30 | 20 | 68 | 11 | 51 | 46 | 42 | 30 | 32 | 66 | 22 | 39 | 23 | - | - | - | - | - | - | - | - | - | - | - | - |
| Pang [[24](#_ENREF_24)] | 36 | 27 | 33 | 30 | 26 | 37 | 31 | 32 | 48 | 15 | 24 | 39 | 46 | 17 | 22 | 41 | 10 | 53 | 0 | 63 | 26 | 37 | 22 | 41 | - | - | - | - |
| Qiu [[25](#_ENREF_25)] | 23 | 37 | 21 | 39 | 19 | 41 | 32 | 28 | 38 | 22 | 31 | 29 | - | - | - | - | - | - | - | - | 29 | 31 | 24 | 36 | 42 | 18 | 32 | 28 |
| Wang [[48](#_ENREF_48)] | 14 | 6 | 15 | 5 | 4 | 16 | 5 | 15 | - | - | - | - | - | - | - | - | - | - | - | - | - | - | - | - | - | - | - | - |
| Wang [[56](#_ENREF_56)] | 16 | 19 | 10 | 10 | 20 | 15 | 9 | 11 | 24 | 11 | 6 | 14 | - | - | - | - | 26 | 9 | 5 | 15 | - | - | - | - | - | - | - | - |
| Wang [[65](#_ENREF_65)] | 7 | 8 | 4 | 11 | 10 | 5 | 10 | 5 | 12 | 3 | 5 | 10 | 12 | 3 | 4 | 11 | - | - | - | - | 10 | 5 | 6 | 9 | - | - | - | - |
| Wu [[66](#_ENREF_66)] | 20 | 16 | 3 | 3 | - | - | - | - | 17 | 19 | 4 | 2 | 31 | 5 | 0 | 6 | - | - | - | - | 36 | 0 | 1 | 5 | - | - | - | - |
| Xiong [[57](#_ENREF_57)] | - | - | - | - | 8 | 21 | 11 | 18 | - | - | - | - | 22 | 6 | 16 | 14 | 8 | 20 | 1 | 29 | 10 | 19 | 8 | 21 | - | - | - | - |
| Xu [[31](#_ENREF_31)] | 12 | 21 | 33 | 69 | - | - | - | - | - | - | - | - | 8 | 25 | 54 | 48 | - | - | - | - | 8 | 25 | 19 | 86 | - | - | - | - |
| Yang [[46](#_ENREF_46)] | 31 | 21 | 27 | 25 | - | - | - | - | 25 | 27 | 11 | 41 | 31 | 21 | 12 | 40 | - | - | - | - | 22 | 30 | 21 | 31 | - | - | - | - |
| Yao [[26](#_ENREF_26)] | 31 | 72 | 12 | 22 | 33 | 70 | 10 | 24 | - | - | - | - | 51 | 52 | 13 | 21 | - | - | - | - | 28 | 75 | 4 | 30 | - | - | - | - |
| Zhang [[34](#_ENREF_34)] | 23 | 23 | 26 | 34 | 20 | 26 | 28 | 32 | 30 | 16 | 11 | 49 | 13 | 33 | 6 | 55 | 5 | 41 | 9 | 51 | 14 | 32 | 25 | 35 | - | - | - | - |
| Zhang [[53](#_ENREF_53)] | 26 | 16 | 21 | 14 | 16 | 22 | 23 | 16 | 11 | 35 | 6 | 25 | 12 | 35 | 18 | 12 | - | - | - | - | 18 | 19 | 26 | 14 | - | - | - | - |
| Zhang [[54](#_ENREF_54)] | 13 | 21 | 7 | 19 | 24 | 10 | 14 | 12 | - | - | - | - | 26 | 8 | 13 | 13 | - | - | - | - | 15 | 19 | 14 | 12 | 24 | 10 | 10 | 16 |
| Zheng [[47](#_ENREF_47)] | 22 | 51 | 16 | 57 | 20 | 53 | 37 | 36 | - | - | - | - | 42 | 31 | 49 | 24 | - | - | - | - | 17 | 56 | 16 | 57 | 42 | 31 | 49 | 24 |
| Zuo [[13](#_ENREF_13)] | 13 | 8 | 11 | 11 | - | - | - | - | 14 | 7 | 11 | 11 | 12 | 9 | 5 | 17 | 8 | 13 | 2 | 20 | - | - | - | - | - | - | - | - |

a1, a0 (ref): the number of MALAT1 overexpression; b1, b0 (ref): the number of normal/low expression of MALAT1.
